# An Improved ID-Based Data Storage Scheme for Fog-Enabled IoT Environments

**DOI:** 10.3390/s22114223

**Published:** 2022-06-01

**Authors:** Han-Yu Lin, Tung-Tso Tsai, Pei-Yih Ting, Ching-Chung Chen

**Affiliations:** Department of Computer Science and Engineering, National Taiwan Ocean University, Keelung 202, Taiwan; tttsai@mail.ntou.edu.tw (T.-T.T.); pyting@mail.ntou.edu.tw (P.-Y.T.); 10857027@email.ntou.edu.tw (C.-C.C.)

**Keywords:** ID-based, data storage, proxy re-encryption, fog computing, IoT

## Abstract

In a fog-enabled IoT environment, a fog node is regarded as the proxy between end users and cloud servers to reduce the latency of data transmission, so as to fulfill the requirement of more real-time applications. A data storage scheme utilizing fog computing architecture allows a user to share cloud data with other users via the assistance of fog nodes. In particular, a fog node obtaining a re-encryption key of the data owner is able to convert a cloud ciphertext into the one which is decryptable by another designated user. In such a scheme, a proxy should not learn any information about the plaintext during the transmission and re-encryption processes. In 2020, an ID-based data storage scheme utilizing anonymous key generation in fog computing was proposed by some researchers. Although their protocol is provably secure in a proof model of random oracles, we will point out that there are some security flaws inherited in their protocol. On the basis of their work, we further present an improved variant, which not only eliminates their security weaknesses, but also preserves the functionalities of anonymous key generation and user revocation mechanism. Additionally, under the Decisional Bilinear Diffie–Hellman (DBDH) assumption, we demonstrate that our enhanced construction is also provably secure in the security notion of IND-PrID-CPA.

## 1. Introduction

According to the concept of cloud computing addressed by computer scientist John McCarthy [1] in 1992, the computing capability of computers will someday become a kind of public utility like telephone systems. Essentially, the cloud computing is an innovative computing concept, rather than a brand-new technique. It utilizes the network to provide the service of data computing, transmitting and sharing. Moreover, it allows lots of computers to simultaneously share the same computing task for not only improving the efficiency, but also solving the plight of insufficient hardware resources in a single computer. From the perspective of end users, they only need to focus on the required resources and service types. Generally speaking, there are three types of service models described as follows:

(i) Software as a Service (SaaS): Users can utilize the browser of information devices such as computers, cell phones, tablets and so on to access the resources and services of cloud providers and execute the required software and applications in highly malleable cloud infrastructures.
(ii) Platform as a Service (PaaS): The cloud service provider offers the platform for application development and the supported programming language with development tools so that users can deploy or purchase the required application services by themselves.
(iii) Infrastructure as a Service (IaaS): In the cloud infrastructure, cloud service providers offer all kinds of resources, including network, storage, analysis and computing, etc., so that users process tasks as if they were on the local machine without maintaining and managing the backend hardware structure.

Though the notion of cloud computing has greatly changed the traditional way of information utilization, sharing and storage, its high latency caused by the Internet transmission and the centralized processing burden of cloud systems are still major challenges in current IoT-enabled cloud applications. Owing to this reason, the model of fog computing has thus come up. It can be regarded as a technique expanded from the cloud computing. Moreover, it is more like a computing mode that is close to the end-users. Therefore, we could say that a fog is a kind of cloud approaching the ground. As compared with clouds, though fogs have a less powerful computing power, they could reduce response time, gain more energy savings and decrease the utilization of bandwidths. So far, fog computing approaches have been used in harsh operational environments such as shipping [2] and aviation [3].

**Figure 1:**
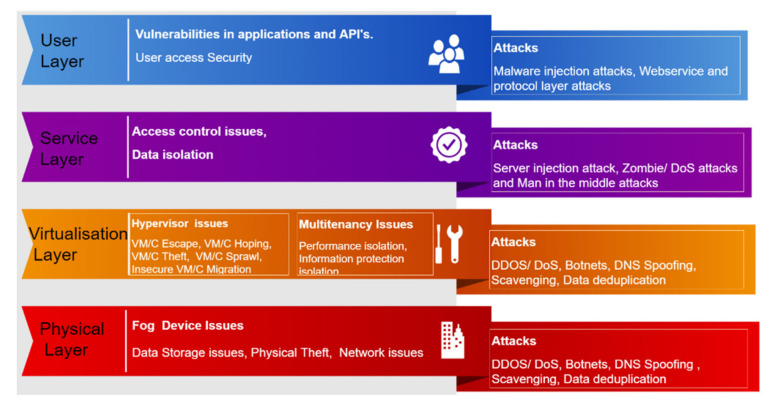
Security threats of fog computing [4].

In a fog-enabled IoT environment, fog nodes are core components that could be either physical devices or virtual equipment, and tightly coupled with intelligent terminals or the access network to provide computing resources. These fog nodes can forward received data to clouds and help with the downloading of user data. Like cloud databases, the fog layer has its own data storage and maintains the local database. Although the architecture of fog computing extended from that of cloud computing can increase mobility and reduce transmission latency, it is still vulnerable to many security threats summarized by Patwary et al. [4] in Figure 1. 

When it comes to sharing confidential data in fog computing, the proxy re-encryption (abbreviated to PRE) scheme addressed by Blaze et al. [5] is a relevant alternative, since it can maintain data confidentiality during the transformation of ciphertexts. Specifically, a data owner can first encrypt the data and then upload the ciphertext to clouds for future access. As the data are transmitted and stored in the encrypted form, anyone including the honest-but-curious cloud server is unable to decrypt it without knowing the corresponding private key. When a data owner attempts to grant another user the access right of his cloud ciphertext, he could authorize a semi-trusted fog node (viewed as the proxy) to perform the procedure of ciphertext re-encryption. In such a way, an original cloud ciphertext is converted into the one that is decryptable by the designated data user. A major advantage of the technique of PREs is that the ciphertext remains undecrypted during the conversion process. Consequently, the proxy will learn nothing about the ciphertext. Up to the present, it has been found in many of the PRE applications [5,6,7,8] such as data sharing, data outsourcing, data storage in clouds, e-mail forwarding, etc. 

### 1.1. Related Works

Under the Decisional Bilinear Diffie–Hellman (DBDH) assumption, in 2005, Ateniese et al. [6,7] presented an improved PRE scheme following the work of Dodis and Ivan [9]. They demonstrated that PRE schemes are useful in the access control of secure file systems and could be realized efficiently in practice. In 2007, Caneti and Hohenberger [10] proposed the definition of chosen-ciphertext attacks (CCA) for PRE schemes and gave a concrete construction to satisfy the definition in the standard model. The definition that they introduced includes both game-based and simulation-based ones. The underlying security of their scheme is also the DBDH assumption. 

Seeing that previous PRE schemes mainly convert the ciphertext from one public key to another, Green and Ateniese [11] introduced identity-based PRE (abbreviated to IB-PRE) schemes to deal with the problem of transforming the ciphertext from one identity to another. In addition, their schemes are non-interactive and provably secure in the random oracle models. One of their works also exhibits the property of multi-hop, i.e., a ciphertext could be converted from one identity to another more than once, which gives the practical applications more feasibility. Using standard model proofs, Chu and Tzeng [12] presented two identity-based PRE mechanisms. They showed that their first scheme has better efficiency in computational costs and the ciphertext length while the other one achieves CCA security. The two mechanisms are unidirectional and non-interactive. Nevertheless, in 2009, Shao and Cao [13] pointed out that the Chu-Tzeng scheme is not truly CCA-secure, as its re-encrypted ciphertext could be further converted into a well-formed ciphertext. Using the Decisional Diffie–Hellman (DDH) assumption and the integer factorization assumption, they also addressed a new PRE scheme that could withstand both the chosen-ciphertext attack and the collusion attack in the random oracle models.

In 2012, Fang et al. [14] presented the so-called conditional PRE with keyword search (C-PRES), which is a combination of conditional PRE (C-PRE) and public key encryption with keyword search (PEKS). They defined the security of chosen-ciphertext attacks for C-PRES and proved that their construction fulfills this definition. Wang et al. [15] also devoted their attention to the research of PRE with keyword search and hence introduced a new primitive named constrained single-hop unidirectional PRE supporting conjunctive keywords search (CPRE-CKS). Based on Park et al.’s work [16], Wang et al.’s scheme only allows the ciphertext containing the corresponding keywords to be re-encrypted by a proxy. Under the decisional *q*-parallel bilinear Diffie–Hellman exponent assumption, in 2013, Liang et al. [17] extended the traditional PRE into the CP-ABPRE, i.e., ciphertext policy-attribute-based PRE. In such a protocol, a proxy has the ability to re-encrypt the ciphertext from one access policy to another. Their protocol can be applied to any monotonic access structure and is proved secure in the random oracle models.

Akhil et al. [18] employed the technique of PRE to enhance the security of QR codes in 2016. A QR code is a machine-readable format that could be tampered with maliciously when being transmitted. Applying the technique of PREs to QR codes makes it easy and flexible to be shared and stored among different hosts. In 2018, Zeng and Choo [19] proposed a new kind of conditional PRE (C-PRE) scheme called sender-specified PRE (SS-PRE) in which a proxy can only convert the ciphertext from a specified sender to his/her delegatee. They also demonstrated that their SS-PRE scheme outperforms the conventional C-PRE ones.

Considering the communication security between the fog and the cloud, in 2018, Vohra and Dave [20] proposed an attribute-based access control and re-encryption system composed of two phases. In the first phase, the clouds will communicate with the proxy server and transmit encrypted data, which are then decrypted by the latter according to its attribute set and access policy. In the second phase, the proxy server will broadcast re-encrypted ciphertext to all fog nodes. Only the fog node that has the correct attributes can decrypt the ciphertext. 

In 2020, Lian et al. [21] introduced a PRE model along with a concrete scheme that is suitable for complex access control factor description in hybrid clouds. A hybrid cloud has not only the advantage of more powerful computing resources in public clouds, but also that of easy management in private clouds. They showed that their construction is secure under the DBDH assumption and could be reliably deployed in hybrid clouds. 

In 2021, Xiong et al. [22] proposed an adaptively secure puncturable identity-based PRE scheme for securing group messages. In their work, a message server is responsible for converting the ciphertext for each user, and thus the heavy computation load could be shifted from the user to the message server. They also prove the security of their system under the DBDH assumption. However, the message server would easily become the performance bottleneck, and the centralized architecture is not suitable for distributed application environments. 

Considering the data sharing in clouds, Ge et al. [23] presented a verifiable and fair attribute-based PRE scheme in which the user is able to verify the correctness of the ciphertext converted by the cloud server. Moreover, the latter is also capable of claiming its honesty when being maliciously accused by the former. They conduct experiments to demonstrate the feasibility and the efficiency of their system in the realistic environments. Nevertheless, their mechanism does not deal with the revocation issue of either attributes or the user identity.

Recently, Zhang et al. [24] applied the technique of PRE to propose an ID-based data storage (DS) system utilizing anonymous key generation for the fog computing environment. A DS scheme for fog-enabled environments is a kind of data sharing technique utilizing fog nodes as the proxy to reduce response time and communication overheads. Specifically, the fog nodes can process the data gathered by the IoT sensors and forward them to the cloud. The fog node serving as a proxy between the cloud and the endpoints is able to transform the cloud ciphertext into another one, which is decryptable by the requested user, so as to achieve the purpose of data sharing in clouds. Using anonymous key generation, a malicious private key Generation (PKG) center is unable to learn the genuine private key of users. It has been formally proved that their protocol is indistinguishable against adaptively chosen identity and chosen plaintext attacks (abbreviated to IND-PrID-CPA) in random oracle models and secure against the PKG and the collusion attacks. However, in this work, we will show that their system has several security flaws. So far, lots of PRE-related cryptographic protocols [25,26,27,28,29,30,31,32,33,34,35,36] have been proposed. 

### 1.2. Contributions

Since a secure DS scheme in fog-enabled IoT environments is the key to ensure data confidentiality and user privacy, we devote ourselves to the enhancement of current DS schemes. 

In 2020, Zhang et al. [24] proposed a novel DS scheme supporting anonymous key generation, which is unnecessary to rely on a secure channel. Unfortunately, their system is vulnerable to several attacks. Motivated by Zhang et al.’s work [24], we present an improved DS scheme in the fog-enabled IoT environments. The novelty of our work is that we view fog nodes as semi-trusted entities rather than fully trusted ones in our system. Moreover, we introduce an additional random value in generating the proxy re-encryption keys, so as to prevent unauthorized decryption. The partial private key information will not be compromised during communication, which gains more protection of confidentiality in practical environments. Our work can strengthen the application security in fog-enabled environments. In particular, telemedicine has received much attention in recent years. The data confidentiality and user privacy are the most concerned. Improving existing schemes to withstand possible attacks is of utmost importance. The major contributions of this research are itemized below:

(i) We demonstrate some security vulnerabilities in relation to the illegal access privilege of Zhang et al.’s scheme [24], including the proxy attack, the outsider attack and the revoked user attack.
(ii) An enhanced DS variant on the basis of Zhang et al.’s system [24] is further proposed. In particular, we introduce an additional random value in the re-encryption key generation and modify the decryption algorithm.
(iii) We formally prove that the proposed construction is indistinguishable against adaptively chosen identity and chosen plaintext attacks (IND-PrID-CPA) in random oracle models.
(iv) The superior characteristics of anonymous key generation and user revocation are preserved in the proposed improvement.
(v) The confidentiality of partial private key information is well-protected during communication since it does not need to be transmitted with the communication data.

The organization of this paper is described as follows. In Section 2, the computational background and cryptographic assumptions are introduced. We review and analyze Zhang et al.’s protocol [24] in Section 3. A corresponding improvement is also presented. In Section 4, using random oracle models, we define the security notion of IND-PrID-CPA and formally prove the security of our improved variant. Finally, a conclusion is summarized in Section 5. 

## 2. Preliminaries

We describe the property of bilinear pairing and a related computational assumption, which the proposed scheme is based on in this section.

*Definition of Bilinear Pairing*

We let both notions of ***G***_1_ and ***G***_2_ be multiplicative groups and they have the same prime order *p*. A symmetric bilinear pairing is defined as *e*: ***G***_1_ × ***G***_1_ → ***G***_2_. Some characteristics of bilinear pairings are stated below:

(i) Bilinearity

Letting *P* be an element of ***G***_1_ and *x*, *y* be arbitrary integers of *Z_p_*, the equality *e*(*P^x^*, *P^y^*) = *e*(*P*, *P*)*^xy^* will hold.

(ii) Non-degeneracy

There exists *P*, *W* ∈ ***G***_1_^2^, satisfying the inequality *e*(*P*, *W*) ≠ 1.

(iii) Computability

There exists an algorithm that could efficiently compute *e*(*P*, *W*), where *P*, *W* ∈ ***G***_1_^2^.

*Decisional Bilinear Diffie-Hellman (DBDH) Problem*

Given elements (*g*, *g^f^*, *g^s^*, *g^k^*, *e*(*g*, *g*)*^fsk^*, *δ*), where *g*, *g^f^*, *g^s^*, *g^k^* ∈ ***G***_1_^4^ and *e*(*g*, *g*)*^fsk^*, *δ* ∈ ***G***_2_^2^, the DBDH problem is to determine whether *e*(*g*, *g*)*^fsk^* equals to *δ* or not.

*Decisional Bilinear Diffie-Hellman (DBDH) Assumption*

The DBDH assumption holds provided that the advantage for arbitrary probabilistic adversary running in polynomial time, and breaking the DBDH problem is negligible.

## 3. Proposed ID-Based Data Storage Scheme

We first formalize the algorithms of ID-based data storage (abbreviated as IB-DS) schemes and then review Zhang et al.’s work [24]. Several security weaknesses of their scheme will be demonstrated, and a corresponding enhanced variant shall be introduced later.

### 3.1. System Architecture

**Figure 2:**
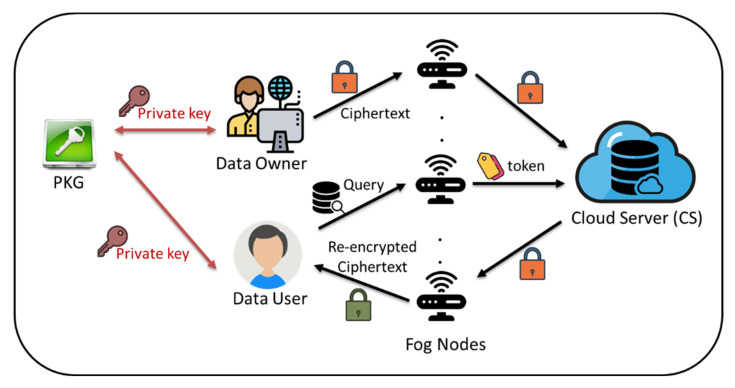
System architecture of IB-DS scheme.

The system architecture of the IB-DS scheme is illustrated in Figure 2, which could be divided into three layers, i.e., the cloud, the fog and the user layers. There is also a trusted authority called the private key generation center (PKG), which is responsible for generating the private key of all involved entities. The cloud server of the cloud layer will store encrypted data gathered from the user layer. A data requester of the user layer can request the data access of the cloud ciphertext by the assistance of the fog layer. The fog nodes comprising the fog layer are viewed as a proxy between the cloud layer and the user layer. Whenever a data owner authorizes the access privilege of a cloud ciphertext to another data user, the proxy (fog) would be granted a re-encryption key, which is able to transform the target cloud ciphertext into one decryptable by the desired data user.

### 3.2. Algorithms

An IB-DS scheme consists of seven algorithms including Setup, Keygen, Encrypt, Query, Permission, Re-encrypt and Decrypt. The definitions of the above algorithms are described below:

- **Setup**(1*^l^*): It accepts a security value *l* and then generates system public parameters *PP* and a master secret key *Msk*.
- **Keygen**(*PP*, *Msk*, *ID*): It takes the input of public parameters *PP*, the master secret key *Msk* and a user identity *ID*, and then outputs the private key *SK_ID_* for the user *ID* via an interactive procedure.
- **Encrypt**(*PP*, *ID*, *m*, *Y*): This algorithm inputs system public parameters *PP*, a user identity *ID*, a message *m* and a symmetric key *Y*, and then outputs the ciphertext *C* of the message *m*.
- **Query**(*PP*, *ID_u_*, *SK_ID__u_*, *M_cate_*): It takes the input of system public parameters *PP*, a data user identity *ID_u_*, a private key *SK_ID__u_* and a data category name *M_cate_*, and then outputs a corresponding query token *TK*.
- **Permission**(*PP*, *ID_u_*, *SK_ID__o_*, *TK*): It takes the input of system public parameters *PP*, a data user identity *ID_u_*, the private key *SK_ID__o_* of the data owner and a query token *TK*, and then outputs either an invalid symbol ⊥ or a re-encryption key *RK*.
- **Re-encrypt**(*PP*, *ID_u_*, *C*, *RK*): It takes the input of system public parameters *PP*, a data user identity *ID_u_*, a ciphertext *C* and a re-encryption key *RK*, and then outputs a corresponding re-encrypted ciphertext *C′*.
- **Decrypt**(*PP*, *SK_ID_*, *C* or *C′*): It takes the input of system public parameters *PP*, a private key *SK_ID_* and a ciphertext *C* (or *C′*), and then outputs a decrypted message *m*.

We summarize the input and the output parameters of each algorithm in Table 1.

### 3.3. Review and Security Analysis

This subsection reviews an IB-DS scheme proposed by Zhang et al. [24] in 2020. Although their protocol is provably secure in the random oracle models, there are still some security drawbacks, which will be pointed out later. The construction of their scheme is described below:

- **Setup:** Using a security value *l*, the PKG first decides two multiplicative groups ***G***_1_ and ***G***_2_. Let *p* be the prime order of both groups and *g* a generator of ***G***_1_. In the two groups, there is a symmetric pairing function *e* expressed as *e*: ***G***_1_ × ***G***_1_ → ***G***_2_. The PKG then chooses integers *a*, *b* ∈ Z_p_* as the *Msk* and computes the *Mpk* = (*P* = *g^a^*, *Q* = *g^b^*). To maintain the membership of system users, the PKG also keeps a revocation list *L*. The system public parameter *PP* is composed of {***G***_1_, ***G***_2_, *e*, *g*, *p*, *Mpk*, *E*(·), *D*(·), *h*_1_, *h*_2_} where *E*(·)/*D*(·) is a symmetric encryption/decryption function and (*h*_1_, *h*_2_) are two secure one-way hash functions that accept a variable-length input and generate a corresponding output in ***G***_1_.
- **Keygen:** A user associated with the identity *ID_i_* first chooses *t_i_*, *z_i_* ∈ Z_p_* to compute
  *Z_i_* = *g^z_i_^*, (1)
  *T′_i_* = *Z_i_* · *h*_1_(*ID_i_* || *t_i_*), (2)
  and transmits (*ID_i_*, *T′_i_*) to the PKG who then chooses *d_i_* ∈ Z_p_* to compute
  *SK′*_*i*,1_ = *g^ab^*(*T′_i_* · *h*_2_(*ID_i_* || *ID_PKG_*))*^d_i_^*, (3)
  *SK′*_*i*,2_ = *g^d_i_^*, (4)
  and delivers (*SK′_i,_*_1_, *SK′_i,_*_2_) to *ID_i_*. In this way, *ID_i_* could further set
  *SK*_*i*,1_ = *SK′*_*i*,1_/(*SK′*_*i*,2_)*^z_i_^* = *g^ab^*(*h*_1_(*ID_i_* || *t_i_*) · *h*_2_(*ID_i_* || *ID_PKG_*))*^d_i_^*, (5)
  *SK*_*i*,2_ = *SK′*_*i*,2_. (6)
  Here, the full private key of *ID_i_* is *SK_i_* = (*SK_i,_*_1_, *SK_i,_*_2_). The correctness of the private key could be verified by the following equality:
  *e*(*SK_i,_*_1_, *g*) = *e*(*P*, *Q*)*e*(*h*_1_(*ID_i_* || *t_i_*) · *h*_2_(*ID_i_* || *ID_PKG_*), *SK_i,_*_2_). (7)
- **Encrypt:** To encrypt the message *m* = (*m*_1_, *m*_2_, …, *m_n_*), a data owner *ID_o_* first selects *r* ∈ _R_ Z_p_* and a symmetric key *Y* ∈ ***G***_2_ to compute
  *α* = *Y* · *e*(*P*, *Q*)*^r^*, (8)
  *β* = *g^r^*, (9)
  *θ* = (*h*_1_(*ID_o_* || *t_o_*) · *h*_2_(*ID_o_* || *ID_PKG_*))*^r^*, (10)
  *τ* = (*E*(*Y*, *m*_1_), *E*(*Y*, *m*_2_), …, *E*(*Y*, *m_n_*)). (11)
  Then, the ciphertext *C* = (*α*, *β*, *θ*, *τ*) along with (*ID_o_*, *M_cate_*), where *M_cate_* represents the category name of data, are transmitted to the nearby fog (proxy), which will keep (*ID_o_*, *M_cate_*, *α*, *β*, *θ*) in the local database of the fog layer and forward (*ID_o_*, *M_cate_*, *τ*) to the cloud server.
- **Query:** To request the data access of *M_cate_*, a data user *ID_u_* first chooses *w* ∈ _R_ Z_p_* to compute
  *W* = (*SK_u,_*_1_)*^w^*, (12)
  and sends (*ID_u_*, *M_cate_*, *W*, *SK_u,_*_2_) to the nearby proxy. Afterwards, the proxy utilizes *M_cate_* to search for matched (*ID_o_*, *M_cate_*, *α*, *β*, *θ*) in the local database and delivers the query token *TK* = (*ID_u_*, *W*, *SK_u,_*_2_, *β*) to the corresponding data owner *ID_o_*.
- **Permission:** When receiving the query token *TK* = (*ID_u_*, *W*, *SK_u,_*_2_, *β*), the data owner sends (*ID_u_*, *SK_u,_*_2_) to the PKG, which will inspect whether *ID_u_* is a revoked user or not according to its revocation list *L* and then return True/False to indicate that the membership of *ID_u_* is valid/invalid. If False, the data owner submits an invalid symbol ⊥ to the proxy. Otherwise, *ID_o_* picks a random number *x* ∈ Z_p_* to compute
  *RK*_1_ = (*SK_o,_*_1_)*W*^−1^*g^x^*, (13)
  *RK*_2_ = *β^x^*, (14)
  *RK*_3_ = *SK*_*o*,2_. (15)
  Then, the re-encryption key *RK* = (*RK*_1_, *RK*_2_, *RK*_3_) is transmitted to the proxy.
- **Re-encrypt:** Given the re-encryption key *RK* = (*RK*_1_, *RK*_2_, *RK*_3_), the proxy first uses the identity *ID_o_* to retrieve *τ* from the cloud server and then computes
  *α′* = *α* · *e*(*RK*_2_, *g*), (16)
  *η* = *RK*_1_, (17)
  *ρ* = *RK*_3_. (18)
  Finally, the resulting ciphertext *C′* = (*α′*, *β*, *θ*, *τ*, *η*, *ρ*) would be returned to the requested data user *ID_u_*.
- **Decrypt:** Given an original ciphertext *C* = (*α*, *β*, *θ*, *τ*), the data owner *ID_o_* first computes
  (19) $$Y=\alpha \cdot \frac{e\left(SK_{o,2},\;\theta \right)}{e\left(SK_{o,1},\;\beta \right)}$$
  and then recovers the message *m* as
  *m* = (*m*_1_, *m*_2_, …, *m_n_*) = (*D*(*Y*, *τ*_1_), *D*(*Y*, *τ*_2_), …, *D*(*Y*, *τ_n_*)). (20)
  We show that Equation (19) correctly derives the symmetric key *Y*. From the right-hand side of the equality, we have
  $$\begin{array}{ccc} \alpha \cdot \frac{e\left(SK_{o,2},\;\theta \right)}{e\left(SK_{o,1},\;\beta \right)} & = & Ye{\left(P,\;Q\right)}^{r}\cdot \frac{e(g^{d_{O}},\;{\left(h_{1}(ID_{O}||t_{O})h_{2}(ID_{O}||ID_{PKG})\right)}^{r})}{e(g^{ab}{\left(h_{1}(ID_{O}||t_{O})h_{2}(ID_{O}||ID_{PKG})\right)}^{d_{O}},\;g^{r})}\\ & = & Ye\left(g^{ab},\;g^{r}\right)\cdot \frac{e(g^{d_{O}},\;{\left(h_{1}(ID_{O}||t_{O})h_{2}(ID_{O}||ID_{PKG})\right)}^{r})}{e(g^{ab}{\left(h_{1}(ID_{O}||t_{O})h_{2}(ID_{O}||ID_{PKG})\right)}^{d_{O}},\;g^{r})}\\ & = & Y \end{array}$$
  When given a ciphertext *C′* = (*α′*, *β*, *θ*, *τ*, *η*, *ρ*) of re-encrypted forms, *ID_u_* computes a symmetric key
  (21) $$Y=\alpha^{\prime }\cdot \frac{e\left(\theta ,\;\rho \right)}{e\left({\left(SK_{u,1}\right)}^{w}\eta ,\;\beta \right)}$$
  and then recovers the message *m* with Equation (20). The correctness of Equation (21) could be verified as follows. From the right-hand side of the equality, we find
  $$\begin{array}{ccc} \alpha^{\prime }\cdot \frac{e\left(\theta ,\;\rho \right)}{e({\left(SK_{u,1}\right)}^{w}\eta ,\;\beta )} & = & Ye{\left(P,\;Q\right)}^{r}e\left(\beta^{x},\;g\right)\cdot \frac{e({\left(h_{1}(ID_{O}||t_{O})h_{2}(ID_{O}||ID_{PKG})\right)}^{r},\;g^{d_{O}})}{e(SK_{u,1}{}^{w}\left(SK_{o,1}\right){\left(SK_{u,1}\right)}^{-w}g^{x},\;g^{r})}\\ & = & Ye\left(g^{ab},\;g^{r}\right)e\left(g^{rx},\;g\right)\cdot \frac{e({\left(h_{1}(ID_{O}||t_{O})h_{2}(ID_{O}||ID_{PKG})\right)}^{r},\;g^{d_{O}})}{e(g^{ab}{\left(h_{1}(ID_{O}||t_{O})h_{2}(ID_{O}||ID_{PKG})\right)}^{d_{O}}g^{x},\;g^{r})}\\ & = & Ye\left(g^{ab},\;g^{r}\right)e\left(g^{rx},\;g\right)\cdot \frac{1}{e\left(g^{ab}g^{x},\;g^{r}\right)}\\ & = & Y \end{array}$$

Note that to revoke the membership of a user *ID_i_*, the PKG will update its revocation list *L* as *L′* by adding the new entry (*ID_i_*, *SK_i,_*_2_), i.e., *L′* = *L* ∪ {(*ID_i_*, *SK_i,_*_2_)}. Unfortunately, the authors find out that Zhang et al.’s scheme [24] has several security weaknesses stated as follows:

***Weakness 1: A dishonest fog (proxy) is able to decrypt the ciphertext queried by a data userID_u_ without having the knowledge of corresponding private key.*** According to Equation (12), the private key information *SK_u_*_,1_ is further combined with a random integer *w* chosen by *ID_u_* for computing the symmetric key *Y*. Although the dishonest proxy knows neither the private key *SK_u_*_,1_ nor the secret *w*, it has obtained the combined value *W* = (*SK_u_*_,1_)*^w^* in the Query phase. Therefore, it can also successfully derive the symmetric key *Y* and decrypt the ciphertext queried by *ID_u_*.

***Weakness 2: ******An adversary is able to gain access to any cloud ciphertext without having the corresponding private key.*** More specifically, an adversary first randomly chooses *SK_u_*_,1_, *w* ∈ Z_p_* to compute *W′* = (*SK_u_*_,1_)*^w^* with respect to any *M_cate_* he attempts to access. Since the adversary is not a revoked user in the revocation list *L*, he would receive a re-encrypted ciphertext. Then, based on the decryption equality, i.e., Equations (20) and (21), he could employ the value *W′* to recover the symmetric key *Y* and decrypt the received ciphertext, respectively.

***Weakness 3: A revoked user can impersonate any legitimate user to gain access to any cloud ciphertext without having the corresponding private key.*** Assume that *ID_u_* is a revoked user in the system. This means that the entry (*ID_u_*, *SK_u_*_,2_) has been stored in the revocation list *L* of the PKG. In order to request any ciphertext in the cloud server, *ID_u_* could impersonate any non-revoked user, say *ID_v_*, to issue a query. The procedure is similar to that mentioned in weakness 2. That is, he first randomly chooses *SK_v_*_,1_, *w* ∈ Z_p_* to compute *W″* = (*SK_v_*_,1_)^w^ in relation to any desired *M_cate_*. As the impersonated identity *ID_v_* is still a legitimate user, the attacker would receive a corresponding re-encrypted ciphertext, which is decryptable by his forged value *W″*.

***Weakness 4: The partial information of the user’s private key is compromised during communication.*** According to the procedures and data flows stated in the Query and the Permission phases, the partial private key *SK_u,_*_2_ of the data user *ID_u_* has to be transmitted via an open channel. This undoubtedly leaks the partial private key information out.

### 3.4. Construction of an Improved IB-DS Scheme

According to our cryptanalyses of Zhang et al.’s system [24], we find out that the private key of the requested data user is not properly hidden in the query algorithm, which makes the secret parameter able to be nullified by any malicious entity in the decryption process. Moreover, the decryption equation, i.e., Equation (21), does not integrate with the second private key of the data user, which is also a major problem that has led to previous attacks. To eliminate the security weaknesses of Zhang et al.’s scheme [24], the authors come up with an improved variant without modifying the system architecture and involved parties. In the Setup algorithm of our system, we additionally introduce a new hash function, i.e., *h*_3_: ***G***_2_ → ***G***_1_. Since the processes of Setup, Keygen and Encrypt algorithms are defined the same as those of Zhang et al.’s scheme [24], we formalize them as the following Algorithms 1–3:
**Algorithm 1**. Setup.**Input:** A security value *l***Output:** *PP*, *Msk*1: Decide groups (***G***_1_, ***G***_2_)2: Choose the prime order *p* and a generator *g*3: Choose appropriate hash functions *h*_1_, *h*_2_ and *h*_3_4: Choose a symmetric encryption/decryption function *E*(·)/*D*(·)5: Define the pairing function *e*: ***G***_1_ × ***G***_1_ → ***G***_2_6: (*a*, *b*) ← Z_p_*7: *P* = *g^a^*8: *Q* = *g^b^*9: *Msk* = (*a*, *b*)10: *Mpk* = (*P*, *Q*)11: *PP* = {***G***_1_, ***G***_2_, *e*, *g*, *p*, *Mpk*, *E*(·), *D*(·), *h*_1_, *h*_2_, *h*_3_}12: **return** (*PP*, *Msk*);


**Algorithm 2.** Keygen.**Input:** *PP*, *Msk*, *ID_i_***Output:** The full private key *SK_i_*1: (*t_i_*, *z_i_*) ← Z_p_*2: *Z_i_* = *g^z_i_^*3: *T′_i_* = *Z_i_* · *h*_1_(*ID_i_* || *t_i_*)4: *d_i_* ← Z_p_*5: *SK′_i,_*_1_ = *g^ab^*(*T′_i_*· *h*_2_(*ID_i_* || *ID_PKG_*))*^d_i_^*6: *SK′_i,_*_2_ = *g^d_i_^*7: *SK_i,_*_1_ = *SK′_i,_*_1_/(*SK′_i,_*_2_)*^z_i_^*8: *SK_i,_*_2_ = *SK′_i,_*_2_9: *SK_i_* = (*SK_i,_*_1_, *SK_i,_*_2_)10: **return** *SK_i_*;



**Algorithm 3.** Encrypt.**Input:** *PP*, *ID*, *m*, *Y***Output:** A ciphertext *C*1: *r* ← Z_p_*2: *Y* ← ***G***_2_3: *α* = *Y* · *e*(*P*, *Q*)*^r^*4: *β* = *g^r^*5: *θ* = (*h*_1_(*ID_o_* || *t_o_*) · *h*_2_(*ID_o_* || *ID_PKG_*))*^r^*6: *τ* = (*E*(*Y*, *m*_1_), *E*(*Y*, *m*_2_), …, *E*(*Y*, *m_n_*))7: *C* = (*α*, *β*, *θ*, *τ*)8: Store (*ID_o_*, *M_cate_*, *α*, *β*, *θ*) in the local database.9: Send (*ID_o_*, *M_cate_*, *τ*) to the cloud server.10: **return** *C*;

- **Query:** To request the data access of *M_cate_*, a data user *ID_u_* first chooses *w* ∈ _R_ Z_p_* to compute
  *W* = *g^w^*, (22)
  and sends (*ID_u_*, *M_cate_*, *W*) to the nearby proxy. Afterwards, the proxy utilizes *M_cate_* to search for matched (*ID_o_*, *M_cate_*, *α*, *β*, *θ*) in the local database and delivers the query token *TK* = (*ID_u_*, *W*, *β*) to the corresponding data owner *ID_o_*. The query processes are presented in Algorithm 4.

**Algorithm 4.** Query.**Input:** *PP*, *ID_u_*, *SK_IDu_*, *M_cate_***Output:** A query token *TK* or ⊥1: *w* ← Z_p_*2: *W* = (*SK_u,_*_1_)*^w^*3: Send (*ID_u_*, *M_cate_*, *W*, *SK_u,_*_2_) to the proxy.4: **if** *M_cate_= M_cate_* of the local database **then**5:  *TK* = (*ID_u_*, *W*, *SK_u,_*_2_, *β*)6:  **return** *TK*;7: **else**8:  **return** ⊥;9: **end if**

- **Permission:** When receiving the query token *TK* = (*ID_u_*, *W*, *β*), the data owner sends *ID_u_* to the PKG, which will inspect whether *ID_u_* is a revoked user or not according to its revocation list *L* and then return True/False to indicate that the membership of *ID_u_* is valid/invalid. If False, the data owner submits an invalid symbol ⊥ to the proxy. Otherwise, *ID_o_* picks two random numbers *x*, *π* ∈ Z_p_* to compute (*RK*_2_, *RK*_3_) as Equations (14) and (15), and (*RK*_1_, *RK*_4_) as

(23) $${\mathrm{RK}}_{1}=\frac{SK_{o,1}g^{x}}{h_{3}\left(e\left(PW^{\pi },\;Q\right)\right)}$$

*RK*_4_ = *e*(*g*, *Q^π^*) (24)

Then, *RK* = (*RK*_1_, *RK*_2_, *RK*_3_, *RK*_4_) is the generated re-encryption key, which will be transmitted to the proxy. The permission processes are presented in Algorithm 5.
**Algorithm 5.** Permission.**Input:** *PP*, *ID_u_*, *SK_IDo_*, *TK***Output:** ⊥ or *RK*1: **if** *ID_u_* is revoked **then**2:  **return** ⊥;3: **else**4:  *x*, *π* ∈ Z_p_*5:  *RK*_1_$=\frac{SK_{o,1}g^{x}}{h_{3}\left(e\left(PW^{\pi },\;Q\right)\right)}$
6:  *RK*_2_ = *β^x^*7:  *RK*_3_ = *SK_o,_*_2_8:  *RK*_4_ = *e*(*g*, *Q^π^*)9:  *RK* = (*RK*_1_, *RK*_2_, *RK*_3_, *RK*_4_)10:  **return** *RK*;11: **end if**

- **Re-encrypt:** Given *RK* = (*RK*_1_, *RK*_2_, *RK*_3_, *RK*_4_), the proxy first uses the identity *ID_o_* to retrieve *τ* from the cloud server and then computes (*α′*, *β*, *θ*, *η*, *ρ*) as Equations (9), (10), and (16)–(18), and Φ as

Φ = *RK*_4_. (25)

**Figure 3:**
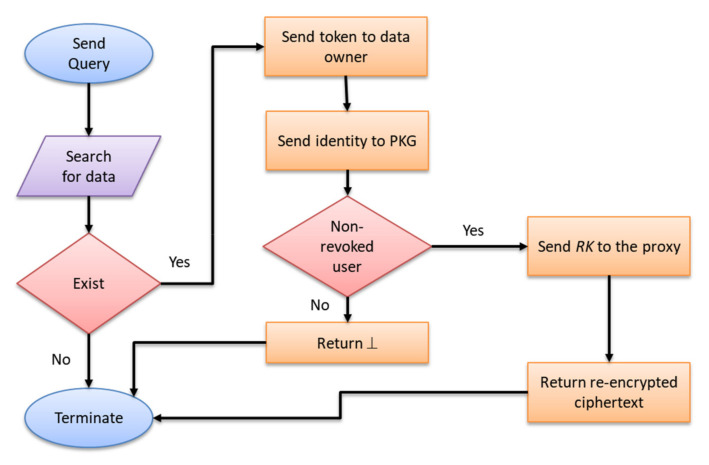
The flow chart of query, permission and re-encryption algorithms.

Finally, the re-encrypted ciphertext *C′* = (*α′*, *β*, *θ*, *τ*, *η*, *ρ*, Φ) is returned to the data user *ID_u_*. We illustrate the flow chart of query, permission and re-encryption algorithms in Figure 3. The re-encryption processes are presented in Algorithm 6.
**Algorithm 6**. Re-Encrypt.**Input:** *PP*, *ID_u_*, *C*, *RK* = (*RK*_1_, *RK*_2_, *RK*_3_, *RK*_4_)**Output:** A re-encrypted ciphertext *C’*1: *α′* = *α* · *e*(*RK*_2_, *g*)2: *β* = *g^r^*3: *θ* = (*h*_1_(*ID_o_* || *t_o_*) · *h*_2_(*ID_o_* || *ID_PKG_*))*^r^*4: *η* = *RK*_1_5: *ρ* = *RK*_3_6: Φ = *RK*_4_7: *C′* = (*α′*, *β*, *θ*, *τ*, *η*, *ρ*, Φ)8: **return** *C′*;

- **Decrypt:** Given an original ciphertext *C* = (*α*, *β*, *θ*, *τ*), the data owner *ID_o_* first computes *Y* as Equation (19) and then recovers the message *m* = (*m*_1_, *m*_2_, …, *m_n_*) = (*D*(*Y*, *τ*_1_), *D*(*Y*, *τ*_2_), …, *D*(*Y*, *τ_n_*)) by Equation (20). Still, when given a re-encrypted ciphertext *C′* = (*α′*, *β*, *θ*, *τ*, *η*, *ρ*, Φ), the data user *ID_u_* first computes *I* and the symmetric key *Y* separately as
  (26) $$\begin{array}{c} I=\eta \cdot h_{3}(\frac{\Phi^{w}e\left(SK_{u,1},\;g\right)}{e\left(h_{1}(ID_{u}||t_{u})h_{2}(ID_{u}||ID_{PKG}),\;SK_{u,2}\right)})\\ =\frac{SK_{o,1}g^{x}}{h_{3}\left(e\left(PW^{\pi },\;Q\right)\right)}h_{3}(\frac{\Phi^{w}e\left(P,\;Q\right)e\left(h_{1}(ID_{u}||t_{u})h_{2}(ID_{u}||ID_{PKG}),\;SK_{u,2}\right)}{e\left(h_{1}(ID_{u}||t_{u})h_{2}(ID_{u}||ID_{PKG}),\;SK_{u,2}\right)})\\ =\frac{SK_{o,1}g^{x}}{h_{3}\left(e\left(PW^{\pi },\;Q\right)\right)}h_{3}\left(e{\left(g,\;Q^{\pi }\right)}^{w}e\left(P,\;Q\right)\right)\\ =SK_{o,1}g^{x} \end{array}$$
  (27) $$Y=\alpha^{\prime }\cdot \frac{e\left(\theta ,\;\rho \right)}{e\left(I,\;\beta \right)}$$
  and then recovers the message *m* with Equation (20). The correctness of Equation (27) could be verified as follows. From the right-hand side of the equality, we find
  $$\begin{array}{c} \alpha^{\prime }\cdot \frac{e\left(\theta ,\;\rho \right)}{e\left(I,\;\beta \right)}\\ =Ye{\left(P,\;Q\right)}^{r}e\left(\beta^{x},\;g\right)\cdot \frac{e({\left(h_{1}(ID_{O}||t_{O})h_{2}(ID_{O}||ID_{PKG})\right)}^{r},\;g^{d_{O}})}{e(\left(SK_{o,1}\right)g^{x},\;g^{r})}\\ =Ye\left(g^{ab},\;g^{r}\right)e\left(g^{rx},\;g\right)\cdot \frac{e({\left(h_{1}(ID_{O}||t_{O})h_{2}(ID_{O}||ID_{PKG})\right)}^{r},\;g^{d_{O}})}{e(g^{ab}{\left(h_{1}(ID_{O}||t_{O})h_{2}(ID_{O}||ID_{PKG})\right)}^{d_{O}}g^{x},\;g^{r})}\\ =Ye\left(g^{ab},\;g^{r}\right)e\left(g^{rx},\;g\right)\cdot \frac{1}{e(g^{ab}g^{x},\;g^{r})}\\ =Y \end{array}$$

Note that to revoke the membership of a user *ID_i_*, the PKG will update its revocation list *L* as *L′* by adding the new entry *ID_i_*, i.e., *L′* = *L* ∪ {*ID_i_*}. The decryption processes are presented in Algorithm 7.
**Algorithm 7.** Decrypt.**Input:** *PP*, *SK_ID_*, *C***Output:** The recovered message *m*1: **if** *C* = (*α*, *β*, *θ*, *τ*) **then**2:  
$Y=\alpha \cdot \frac{e\left(SK_{o,2},\;\theta \right)}{e\left(SK_{o,1},\;\beta \right)}$
3: **elseif** *C* = (*α′*, *β*, *θ*, *τ*, *η*, *ρ*, Φ) **then**4:  
$I=\eta \cdot h_{3}(\frac{\Phi^{w}e\left(SK_{u,1},\;g\right)}{e\left(h_{1}(ID_{u}||t_{u})h_{2}(ID_{u}||ID_{PKG}),\;SK_{u,2}\right)})$
5:  
$Y=\alpha^{\prime }\cdot \frac{e\left(\theta ,\;\rho \right)}{e\left(I,\;\beta \right)}$
6: **end if**7: **for** *i* = 1 to *n*8:  *m_i_* = *D*(*Y*, *τ_i_*)9: **next** *i*10: *m* = (*m*_1_, *m*_2_, …, *m_n_*)11: **return** *m*;

## 4. Security Model and Proof

To formally prove the security of our improved IB-DS scheme, the authors first present its security model and then give a completed security proof. Since the core building block of the IB-DS scheme is actually the IB-PRE scheme, the notion of a security model for the former also comes from that for the latter. Specifically, we will prove that our improved IB-DS construction is indistinguishable against the adaptively chosen identity and chosen plaintext attacks (IND-PrID-CPA). The security model of IND-PrID-CPA for the IB-DS scheme is defined as follows.

**Definition** **1.** (IND-PrID-CPA). *An IB-DS scheme achieves the indistinguishability against adaptively chosen identity and chosen-plaintext attacks if in the following game, there is no probabilistic adversary A who is able to defeat a challenger B with non-negligible advantage in polynomial-time:*

**Setup:** In the beginning, the challenger *B* performs the Setup (1*^l^*) algorithm to initialize the system public parameters *PP* and a master secret key *Msk*. Then, the parameters *PP* are sent to *A*.
**Phase 1:** The adversary *A* will make the following queries adaptively:
  - *Private-key Queries:* In this query, the adversary *A* will provide an identity *ID* for the challenger *B* who then calls the Keygen (*PP*, *Msk*, *ID*) algorithm to get the corresponding private key *SK_ID_* and returns it.
  - *Permission Queries:* In this query, the adversary *A* will provide two identities (*ID_o_*, *ID_u_*) of non-revoked users and a data category name *M_cate_* for the challenger *B* who first calls the Keygen (*PP*, *Msk*, *ID*) algorithm to gain the private keys *SK_IDo_* and *SK_IDu_*. Next, *B* performs the Query (*PP*, *ID_u_*, *SK_ID__u_*, *M_cate_*) and the Permission (*PP*, *ID_u_*, *SK_ID__o_*, *TK*) algorithms to obtain the re-encryption key *RK* and returns it.
**Challenge:** The adversary *A* determines a target identity ID*, a message m* = (*m*_1_*, *m*_2_*, …, m_n_*) and two symmetric keys (*Y*_0_, *Y*_1_) of the same length. Next, the challenger *B* takes the input of (*PP*, ID*, m*, *Y_λ_*) where *λ* ∈ *_R_*{0, 1} to produce a ciphertext C* = (α*, β*, θ*, τ*) as the challenge for *A*.
**Phase 2:** After receiving the challenge, the adversary *A* can further make queries defined as those in phase 1, except for the following restrictions:
  - A private-key query for the target identity ID* is not allowed.
  - Any permission query in relation to the identities of the form (ID*, *ID_u_*) or (*ID_o_*, ID*) is not allowed.
  - The maximum number of times for the private key and the permission queries are bound by *q_pk_* and *q_pr_*.
**Guess:** When phase 2 terminates, the adversary *A* outputs a bit *λ*′. If *λ*′ = *λ*, *A* is the winner of the game. Consequently, the advantage of *A* is defined as *Adv*(*A*) =|Pr[*λ*′ = *λ*] − 1/2|.

On the basis of a previously defined security model, we formally prove that our improved IB-DS construction is IND-PrID-CPA-secure in the proof model of random oracles below.

**Theorem** **1.** (IND-PrID-CPA). *Let h_i_ (for i = 1 and 2) be random oracles. The proposed IB-DS system is indistinguishable against adaptively chosen identity and chosen-plaintext attacks (IND-PrID-CPA) under the DBDH assumption. In particular, if a probabilistic polynomial–time adversary A making at most q_pk_ and q_pr_ queries breaks the IND-PrID-CPA security of our IB-DS scheme with the non-negligible advantage ε, an algorithm B solving the DBDH problem can be constructed with the non-negligible advantage ε′ where*

$$\varepsilon^{\prime }\;\geq \;\frac{\varepsilon }{e(\sqrt{q_{pk}+q_{pr}+1})}.$$

**Figure 4:**
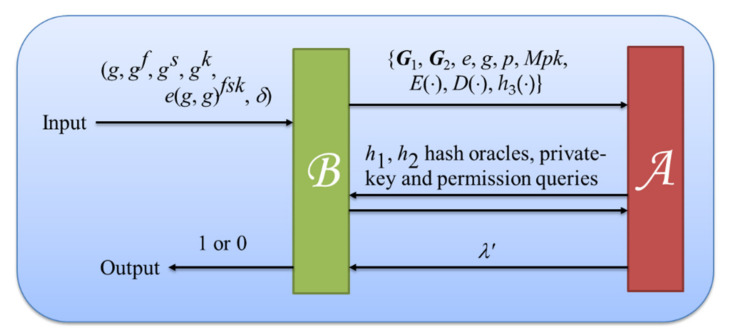
The simulation game between the adversary *A* and the algorithm *B* of Theorem 1.

**Proof.**  We depict the proof structure as Figure 4. Let (*g*, *g^f^*, *g^s^*, *g^k^*, *e*(*g*, *g*)*^fsk^*, *δ*) be a problem instance of DBDH for *B* whose purpose is to decide if *e*(*g*, *g*)*^fsk^* equals to *δ* or not by utilizing the advantage of *A*. In addition, the algorithm *B* also serves as a challenger responding to the queries that *A* makes in the following simulation game.

**Setup:** In the beginning, *B* performs the Setup(1*^l^*) function to initialize public parameters *PP* = {***G***_1_, ***G***_2_, *e*, *g*, *p*, *Mpk*, *E*(·), *D*(·), *h*_3_(·)} where *Mpk* = (*P* = *g^f^*, *Q* = *g^s^*). Note that the *Msk* of the PKG is implicitly defined as (*f*, *s*) which *B* does not know. Moreover, *B* chooses a random integer *rn* ∈ Z_p_*. Then, the parameters *PP* are sent to *A*.
**Phase 1:** The adversary *A* will make the following queries adaptively:
  - *h*_1_(*ID_i_* || *t_i_*) *hash oracle:* For any *h*_1_(*ID_i_* || *t_i_*) query, *B* uses (*ID_i_*, *t_i_*) as the index to searches for a matched entry in the *h*_1_-table named HT1. Otherwise, *B* first chooses a bit *bt*_1_ with Pr[*bt*_1_ = 0] = *ψ* where *ψ* will be determined later. When *bt*_1_ = 0, *B* computes *HO*_1_ = *P^rn^g^s^*^1^ where *s*_1_ ∈ Z_p_*; else, *B* computes *HO*_1_ = *g^s^*^1^. Then, *B* updates HT1 as HT1 ∪ {(*ID_i_*, *t_i_*, *bt*_1_, *s*_1_, *HO*_1_)} and returns the value *HO*_1_ to *A*.
  - *h*_2_(*ID_i_* || *ID_PKG_*) *hash* *oracle:* For any *h*_2_(*ID_i_* || *ID_PKG_*) query, *B* uses the identity *ID_i_* as an index to searches for a matched entry in the *h*_2_-table named HT2. Otherwise, *B* first chooses a bit *bt*_2_ with Pr[*bt*_2_ = 0] = *ψ* where *ψ* will be determined later. When *bt*_2_ = 0, *B* computes *HO*_2_ = *P^rn^g^s^*^2^ where *s*_2_ ∈ Z_p_*; else, *B* computes *HO*_2_ = *g^s^*^2^. Then, *B* updates HT2 as HT2 ∪ {(*ID_i_*, *ID_PKG_*, *bt*_2_, *s*_2_, *HO*_2_)} and returns the value *HO*_2_ to *A*.
  - *Private-key Queries:* For the private-key query of *ID_i_*, *B* first uses the identity *ID_i_* as an index to searches for matched entries (*ID_i_*, *t_i_*, *bt*_1_, *s*_1_, *HO*_1_) and (*ID_i_*, *ID_PKG_*, *bt*_2_, *s*_2_, *HO*_2_) in HT1 and HT2, respectively. (If no such entries exist, *B* will invoke *h*_1_ and *h*_2_ queries on behalf of *A*.) When both *bt*_1_ and *bt*_2_ equal to 1, *B* aborts. In cases where both *bt*_1_ and *bt*_2_ equal to 0, *B* selects *d_i_′* ∈ Z_p_* to compute

$${SK}_{i,1}=Q^{\frac{-(s_{1}+s_{2})}{2rn}}{\left(P^{2rn}g^{s_{1}+s_{2}}\right)}^{{d_{i}}^{\prime }}\;\mathrm{and}\;{SK}_{i,2}=Q^{\frac{-1}{2rn}}g^{{d_{i}}^{\prime }}.$$

In the remaining two cases where the values of *bt*_1_ and *bt*_2_ are reversed, *B* also chooses *d_i_′* ∈ Z_p_* to compute

$${SK}_{i,1}=Q^{\frac{-(s_{1}+s_{2})}{rn}}{\left(P^{rn}g^{s_{1}+s_{2}}\right)}^{{d_{i}}^{\prime }}\;\mathrm{and}\;{SK}_{i,2}=Q^{\frac{-1}{rn}}g^{{d_{i}}^{\prime }}.$$

As a matter of fact, in either of the above forms of private keys, *SK_i,_*_1_ and *SK_i,_*_2_ are well-formed, as shown below. To simplify the derivation, we let *vx* be *s*_1_ + *s*_2_ and *rx* be the value of either 2*rn* or *rn*.

$$\begin{array}{ccc} {SK}_{i,1} & = & Q^{\frac{-vx}{rx}}{\left(P^{rx}g^{vx}\right)}^{d_{i}}\\ & = & Q^{f}{\left(P^{rx}g^{vx}\right)}^{\frac{-s}{rx}}{\left(P^{rx}g^{vx}\right)}^{{d_{i}}^{\prime }}\\ & = & Q^{f}{\left(P^{rx}g^{vx}\right)}^{{d_{i}}^{\prime }-\frac{s}{rx}}\\ & = & g^{fs}{\left(h_{1}(ID_{i}||t_{i})h_{2}(ID_{i}||ID_{PKG})\right)}^{{d_{i}}^{\prime }}\;\mathrm{where}\;d_{i}^{\prime \prime }=d_{i}^{\prime }-\frac{s}{rx}\\ {SK}_{i,2} & = & Q^{\frac{-1}{rx}}g^{{d_{i}}^{\prime }}\\ & = & g^{\frac{-s}{rx}}g^{{d_{i}}^{\prime }}\\ & = & g^{{d_{i}}^{\prime }}\;\mathrm{where}\;d_{i}^{\prime \prime }=d_{i}^{\prime }-\frac{s}{rx} \end{array}$$

Then, the computed private keys (*SK_i,_*_1_, *SK_i,_*_2_) are returned to *A*. It is evident to observe that the returned private keys have the identical distribution as those in the real scheme.

- *Permission Queries:* For any permission query of (*ID_o_*, *ID_u_*, *M_cate_*) where *ID_o_* ≠ *ID_u_* and *ID_u_* is not revoked, *B* first obtains their private keys *SK_ID__o_* and *SK_ID__u_* by the private-key queries and then finds out the corresponding information stored in HT1 and HT2. If any of *ID_o_* and *ID_u_* satisfies the condition that both of *bt*_1_ and *bt*_2_ equal to 1, *B* aborts. Otherwise, *B* selects *w*, *x*, *π* ∈ Z_p_* to compute *W* = *g^w^*, *RK*_1_ = $\frac{SK_{o,1}g^{x}}{h_{3}\left(e\left(PW^{\pi },\;Q\right)\right)}$, *RK*_2_ = *β* *^x^*, *RK*_3_ = *SK_o,_*_2_, *RK*_4_ = *e*(*g*, *Q^π^*) where *β* is the partial ciphertext with respect to *M_cate_.* Here, *RK* = (*RK*_1_, *RK*_2_, *RK*_3_, *RK*_4_) is the derived re-encryption key. Then, *B* returns *RK* to *A*.

**Challenge:** The adversary *A* determines a target identity ID*, a message m* = (*m*_1_*, *m*_2_*, …, m_n_*) and two symmetric keys (*Y*_0_, *Y*_1_) of the same length. Next, the challenger *B* takes the input of (*PP*, ID*, m*, *Y_λ_*) where *λ* ∈*_R_*{0, 1} to produce a ciphertext C* = (α*, β*, θ*, τ*) for *A* by the following steps:
**Step 1**: Without loss of generality, we assume that the corresponding hash queries for ID* have been queried by *A*. If any of *bt*_1_* and *bt*_2_* equals to 0, *B* aborts;
**Step 2**: Otherwise, *B* computes
  α* = *Y_λ_*· *δ*,
  β* = *g^k^*,
  θ* = (*g^k^*)^(s1*^^+ s2*^^)^ = (*h*_1_(ID* || t*) · *h*_2_(ID* || *ID_PKG_*))*^k^*,
  where (*s*_1_*, *s*_2_*) are the corresponding random values in relation to ID* and stored in HT1 and HT2, respectively, and
  τ* = (*E*(*Y_λ_*, *m*_1_*), *E*(*Y_λ_*, *m*_2_*), …, *E*(*Y_λ_*, m_n_*)).

At last, the ciphertext C* = (α*, β*, θ*, τ*) is returned to *A* as a challenge ciphertext.

**Phase 2:** After receiving the challenge C*, the adversary *A* can further make queries such as those in phase 1, except for the restrictions stated in Definition 1.
**Guess:** When phase 2 terminates, the adversary *A* outputs a bit *λ*′. If *λ*′ = *λ*, *B* outputs 1; else, *B* outputs 0. The former stands for that *e*(*g*, *g*)*^fsk^* equals to *δ* while the latter does not.
**Analysis:** According to the steps in the challenge phase, if *e*(*g*, *g*)*^fsk^* = *δ*, the simulated C* would be a valid ciphertext, and hence the advantage of *A* to break our construction is non-negligible, i.e., *Adv*(*A*) = | Pr[*λ*′ = *λ*] − 1/2 |≥ *ε*. On the contrary, if *e*(*g*, *g*)*^fsk^* ≠ *δ*, the adversary *A* has no better advantage in guessing *λ*′, meaning that Pr[*λ*′ = *λ*] = 1/2. Let Pr[Perfect] be the probability of the event that the entire simulation game is perfect without accidental termination. Consequently, we could express the advantage of *B* to break the DBDH problem as
                      | Pr[(*g*, *g^f^*, *g^s^*, *g^k^*, *e*(*g*, *g*)*^fsk^*) = 1] − Pr[(*g*, *g^f^*, *g^s^*, *g^k^*, *δ*) = 1] |       ≥ | (1/2 + *ε*) − 1/2 | ⋅ Pr[Perfect] = *ε* ⋅ Pr[Perfect]

To give a better estimation of Pr[Perfect], we first consider the probability that *B* does not abort in any query simulated above. For convenience, we define some probability events below:

Pr[¬PkQ]: the probability that all private-key queries are perfect without being aborted;
Pr[¬PrQ]: the probability that all permission queries are perfect without being aborted;
Pr[¬Ch]: the probability that the challenge phase is perfect without being aborted.

Since all the above probability events are independent, we know that Pr[Perfect] could be further expressed as Pr[Perfect] = Pr[¬PkQ] · Pr[¬PrQ] · Pr[¬Ch]. In a private-key query, *B* aborts when both *bt*_1_ and *bt*_2_ are related to *ID_i_* equal to 1, i.e., Pr[¬PkQ] ≥ (1 − (1 − *ψ*)^2^)*^qpk^*. Similarly, in a permission query, *B* aborts when both *bt*_1_ and *bt*_2_ are related to *ID_o_* or *ID_u_* equal to 1. Hence, we obtain Pr[¬PrQ] ≥ (1 − (1 − *ψ*)^2^)*^qpr^*. Still, in the challenge phase, *B* aborts if any of *bt*_1_* and *bt*_2_* corresponding to ID* equals to 0. That is, Pr[¬Ch] ≥ (1 − *ψ*)^2^. Combining all of these probability events, we have

Pr[Perfect] ≥ [(1 − (1 − *ψ*)^2^)*^q_pk_^*][(1 − (1 − *ψ*)^2^)*^q_pr_^*](1 − *ψ*)^2^ = [(1 − (1 − *ψ*)^2^)*^q_pk_^*
^+ *q_pr_*^](1 − *ψ*)^2^

When *ψ* = $1-\frac{1}{\sqrt{q_{pk}+q_{pr}+1}}$, the probability of Pr[Perfect] achieves the maximum value $\frac{1}{e(\sqrt{q_{pk}+q_{pr}+1})}$ where *e* is the base of natural logarithm. Accordingly, the advantage for *B* to solve the DBDH problem is calculated as *ε′* ≥ $\frac{\varepsilon }{e(\sqrt{q_{pk}+q_{pr}+1})}$. □

**Theorem** **2.** *The proposed construction is secure against the dishonest fog (proxy) that attempts to learn the plaintext from the ciphertext requested by a data user.*

**Proof.**  In the permission phase of our scheme, a dishonest proxy can obtain the re-encryption key *RK* composed of four subkeys in which *RK*_1_ = $\frac{SK_{o,1}g^{x}}{h_{3}\left(e\left(PW^{\pi },\;Q\right)\right)}$. If the proxy tries to derive the private key $SK_{o,1}$ for decrypting the original ciphertext *C* = (*α*, *β*, *θ*, *τ*), he has to know the two random numbers (*x*, *π*), which are chosen by the data owner. Consequently, he cannot successfully derive $SK_{o,1}$ from the re-encryption key *RK*_1_. Furthermore, if he attempts to learn the plaintext from the re-encrypted ciphertext *C′* = (*α′*, *β*, *θ*, *τ*, *η*, *ρ*, Φ), he will face the difficulty in computing the parameter *I* owing to the lack of the data user’s private keys. □

**Theorem** **3.** *The proposed construction is secure against the malicious or compromised PKG who attempts to gain the access to any cloud ciphertext without having the corresponding private key.*

**Proof.**  In the keygen algorithm of the proposed system, we also adopt the technique of anonymous key generation to issue each user’s private keys. Although the private key *SK_i,_*_2_ is controlled by the PKG, it cannot derive the private key *SK_i,_*_1_ = *g^ab^*(*h*_1_(*ID_i_* || *t_i_*) · *h*_2_(*ID_i_* || *ID_PKG_*))*^di^* without knowing the secret value *t_i_* chosen by the user. As for directly computing *h*_1_(*ID_i_* || *t_i_*) from the received parameter *T′_i_*, the PKG also does not have the correct knowledge of *Z_i_* = *g^zi^*. Without having the full private keys of any user, a malicious or compromised PKG is impossible to decrypt either an original or a re-encrypted ciphertext with Equations (19) and (26). □

**Theorem** **4.** *The proposed construction is secure against any revoked user who attempts to impersonate a legitimate user to gain access to any cloud ciphertext without having the corresponding private key.*

**Proof.**  According to the query algorithm in our scheme, a revoked user *ID_u_* impersonating a non-revoked user *ID_v_* first chooses a random number *w* ∈ _R_ Z_p_* to compute *W* = *g^w^*, and sends (*ID_v_*, *M_cate_*, *W*) to the nearby proxy. Since the transmitted identity is *ID_v_*, it will not be rejected by the PKG in the permission phase. Finally, *ID_u_* will receive a re-encrypted ciphertext *C′* = (*α′*, *β*, *θ*, *τ*, *η*, *ρ*, Φ). However, to decrypt the ciphertext, he needs to know the correct private keys of *ID_v_* in addition to his chosen random number *w*. Without the former, he cannot successfully recover the original message. □

We compare the functionality and security of our improved construction with some previous ones including Han et al.’s (HSM for short) [33], Tang et al.’s (THJ for short) [34], Wang et al.’s (WWM) [35], Matsuo’s (Mat for short) [36] and Zhang et al.’s (ZBW for short) [24] in Table 2. From the table, it is obvious that only the schemes of ZBW and ours support anonymous key generation and user revocation. The schemes of THJ and Mat cannot resist the collusion attack plotted by the proxy and the data user. Except for the proposed construction, all the other compared ones are subject to the malicious (compromised) PKG attack. To sum up, our improved DS variant has better functionality and security among all compared mechanisms.

**Figure 5:**
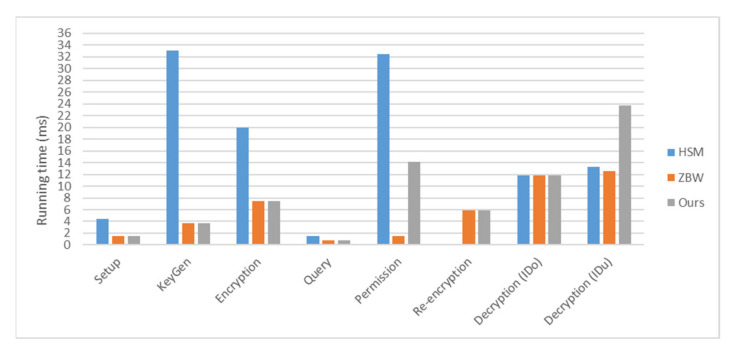
Numerical comparison of computational complexity.

Since only Han et al.’s [33] and Zhang et al.’s schemes [24] have similar structures to ours, we further make the efficiency comparison below. We consider the computation of bilinear pairing and exponentiation in our improved algorithms. The detailed results are shown in Table 3. Note that we use the symbols of “B”, “E1” and “E2” to separately represent a bilinear pairing, an exponentiation computation over ***G***_1_ and an exponentiation computation over ***G***_2_. The numerical comparisons are also illustrated in Figure 5 by using the hardware of Intel Core 2 Duo 2.10 Ghz CPU and 2 GB RAM. The software platform is the Ubuntu 9.10 operating system and the PBC library [37]. The estimated running times of B, E1 and E2 computation are approximately 5.883, 0.736 and 0.142 ms, respectively. Although the proposed algorithms incur higher computational costs in the decryption phase, it could be regarded a reasonable trade-off to obtain a higher security.

**Figure 6:**
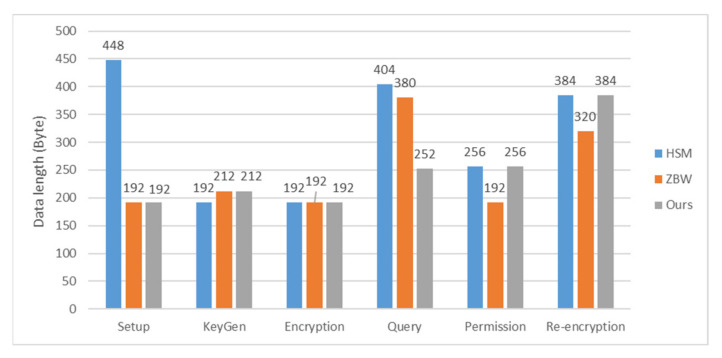
Numerical comparison of communication overheads.

The communication overheads are evaluated in terms of the length of the query token, the ciphertext and the re-encrypted ciphertext. For simplicity, the length of identity and that of the data category name are ignored in the comparison. Assume that the output length of symmetric encryption is |SE|. The detailed results are shown in Table 4. It is evident that the query token length of our scheme is shorter than that of ZBW by |***G***_1_|. Yet, when transmitting the re-encrypted ciphertext, our scheme has to send an extra element of ***G***_2_. We claim that the extra element is crucial for protecting the ciphertext from unauthorized decryption. The numerical comparisons are also illustrated in Figure 6 by using the elliptic curve of embedding degree 2. Hence, the size of order *p* is 160 bits and that of a field element is 512 bits. 

## 5. Conclusions

To enhance the security of more and more popular data applications in fog-enabled IoT environments, in this paper, we proposed an improved data storage scheme following Zhang et al.’s work [24]. We pointed out several security vulnerabilities in their scheme. Concretely speaking, their scheme fails to satisfy the basic access policy that only the user owning the correct private key can decrypt the corresponding cloud ciphertext. Hence, an adversary including the dishonest proxy, malicious PKG and revoked users can arbitrarily request a cloud ciphertext and decrypt it without having the knowledge of corresponding private key. To eliminate the above security flaws, we have modified some algorithms in our improved system. Moreover, we formally proved that our construction is IND-PrID-CPA-secure in the random oracle models. Overall, the advantages of anonymous key generation and user revocation are also preserved in the proposed variant with higher security. Our improved mechanism can provide better security protection for the applications in fog-enabled IoT environments. Although the computational complexity is increased, we believe that it is a worthy trade-off to gain a higher level of security. The limitation of our mechanism is that each user has to maintain an extra secret value chosen at the keygen phase. Such a value will be utilized in the decryption process. The aim of future work should be to combine attribute-based mechanisms for supporting more fine-grained access control policies.

## Figures and Tables

**Table 1:** The input and output parameters of composed algorithms.

	Parameter	Input	Output
Algorithm			
Setup		*l*	*PP*, *Msk*
Keygen		*PP*, *Msk*, *ID*	*SK_ID_*
Encrypt		*PP*, *ID*, *m*, *Y*	*C*
Query		*PP*, *ID_u_*, *SK_ID__u_*, *M_cate_*	*TK* or ⊥
Permission		*PP*, *ID_u_*, *SK_ID__o_*, *TK*	⊥ or *RK*
Re-encrypt		*PP*, *ID_u_*, *C*, *RK*	*C’*
Decrypt		*PP*, *SK_ID_*, *C* or *C′*	*m*

**Table 2:** Comparison of functionality and security.

	Scheme	HSH	THJ	WWM	Mat	ZBW	Ours
Item							
Support anonymous key generation		No	No	No	No	Yes	Yes
Support user revocation		No	No	No	No	Yes	Yes
Withstand the malicious (compromised) PKG		No	No	No	No	No	Yes
Withstand the collusion attack		Yes	No	Yes	No	Yes	Yes
Withstand the dishonest proxy		Yes	Yes	Yes	Yes	No	Yes
Withstand the revoked user		n.a.	n.a.	n.a.	n.a.	No	Yes

Remark: The term of n.a. stands for not applicable.

**Table 3:** Comparison of computational complexity.

	Scheme	HSM	ZBW	Ours
Item				
Cost of setup algorithm		6E1	2E1	2E1
Cost of keygen algorithm		5B + 5E1	5E1	5E1
Cost of encrypt algorithm		3B + 3E1 + E2	B + 2E1 + E2	B + 2E1 + E2
Cost of query algorithm		2E1	E1	E1
Cost of permission algorithm		5B + 4E1 + E2	2E1	2B + 3E1 + E2
Cost of re-encryption algorithm		0	B	B
Cost of decryption algorithm by *ID_o_*		2B	2B	2B
Cost of decryption algorithm by *ID_u_*		2B + 2E1	2B + E1	4B + E2

**Table 4:** Comparison of communication overheads.

	Scheme	ZBW	Ours
Item			
Query token length		3|***G***_1_|	2|***G***_1_|
Ciphertext length		2|***G***_1_| + |***G***_2_| + *n*|SE|	2|***G***_1_| + |***G***_2_| + *n*|SE|
Re-encryption ciphertext length		4|***G***_1_| + |***G***_2_| + *n*|SE|	4|***G***_1_| + 2|***G***_2_| + *n*|SE|
